# R-Group stabilization in methylated formamides observed by resonant inelastic X-ray scattering†

**DOI:** 10.1039/d2cc00053a

**Published:** 2022-07-11

**Authors:** Miguel Ochmann, Vinícius Vaz da Cruz, Sebastian Eckert, Nils Huse, Alexander Föhlisch

**Affiliations:** Department of Physics, University of Hamburg and Center for Free-Electron Laser Science Luruper Chaussee 149 22761 Hamburg Germany nils.huse@uni-hamburg.de; Institute for Methods and Instrumentation in Synchrotron Radiation Research G-ISRR, Helmholtz-Zentrum Berlin für Materialien und Energie Albert-Einstein-Strasse 15 12489 Berlin Germany sebastian.eckert@helmholtz-berlin.de; Institut für Physik and Astronomie, Universität Potsdam Karl-Liebknecht-Strasse 24-25 14476 Potsdam Germany

## Abstract

The inherent stability of methylated formamides is traced to a stabilization of the deep-lying σ-framework by resonant inelastic X-ray scattering at the nitrogen K-edge. Charge transfer from the amide nitrogen to the methyl groups underlie this stabilization mechanism that leaves the aldehyde group essentially unaltered and explains the stability of secondary and tertiary amides.

## Abstract 2

The inherent stability of methylated formamides is traced to a stabilization of the deep-lying σ-framework by resonant inelastic X-ray scattering at the nitrogen K-edge.

## Graphical abstract

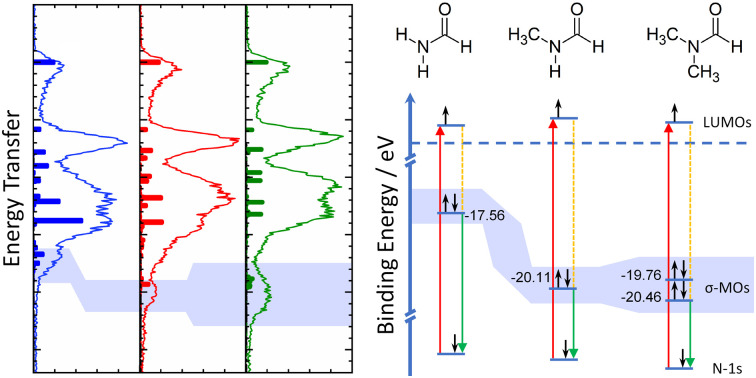

The amide functional group as an important structural motif in organic chemistry and amides are pervasive both, in nature^1^ and technology.^2^ In biochemistry, the amide motif plays a pivotal role in linking amino acids in the protein chain as a peptide bond.^3^ Amides are found in synthetic rubbers with the most prominent representatives being nylons.^4^ Importantly, the amide functional group is very stable due to its low reactivity owing to delocalization of electron density from the nitrogen lone pair into the carbonyl group:^5^ The N-C=O motif contains a delocalized π-system with a partial double-bond character and a planar configuration that hinders the rotation around the C–N bond in contrast to the equivalent oxygen motif, the ester group, which is freely rotatable.^6^ This double-bond character of the amide group creates a conformational constraint in larger, amide-containing molecules that is of particular importance in protein chains and artificial polyamides.

Formamide (FA) is the smallest molecule containing an amide group with many applications from use as a solvent in industrial processes to a precursor of pharmaceutical compounds, also receiving attention as a possible building block in the early development of life.^7^ Methyl substitution of amide hydrogen atoms yields N-Methylformamide (NMF) and *N*,*N*-dimethylformamide (DMF), respectively. These formamide derivatives are part of many applications: NMF is not only used as a solvent but was recently considered as a potential *anti*-tumoral agent while DMF is widely used as solvent for peptide coupling reactions in pharmaceutical research and in synthesis of adhesives, polymers and surface coatings.^8^

The delocalization of electron density from multiple bonds results in a stabilization of the involved molecular orbitals (MOs) and thus, an increase in bond strength. Most prominent is the delocalization of electron density over adjacent p-orbitals, resulting in π-systems and aromaticity with exceptionally strong stabilization of the π-MOs. Similar albeit weaker stabilization is achieved by hyperconjugation – when electron density from a σ-bond is delocalized into a non-bonding p-/antibonding σ*/π*-orbital, or from p/π-orbitals into σ*-orbitals (negative hyperconjugation) – which is usually limited to β atoms in contrast to π-conjugation although there have been some reports on remote and double hyperconjugation.^9^ Hyperconjugation is able to give a quantum mechanical explanation for some steric effects such as the anomeric effect, the gauche effect, the staggered conformation of ethane, and the relative stability of substituted carbocations and carbon centred radicals.^10^

Early studies of *N*-substituted amides suggested stabilization by hyperconjugation if organic groups were bound to the amide nitrogen atom but not until decades later, Wiberg and Breneman showed that the N–C=O π*-system stabilizes the σ-bonds of *N*-unsubstituted amides.^11^ Their studies on the rotational barrier in formamide revealed σ-withdrawal and π donation mediated by the amide nitrogen. In this scheme, the nitrogen atom withdraws electron density from the carboxy carbon atom and donates density *via* its lone pair p-orbital to the C=O π*-orbital. They further concluded that this scheme is most effective in the planar configuration because the amide nitrogen atom's electronegativity strongly decreases upon pyramidalization in the rotation, thereby counteracting the σ-withdrawal and π donation scheme.

In this study, we provide direct experimental evidence from resonant inelastic X-ray scattering (RIXS) for additional stabilization of formamides upon *N*-methylation. Experimental results and theoretical modelling reveal a charge-transfer mechanism in *N*-substituted formamides *via* the deep-lying σ-framework that differs from hyperconjugation of σ-withdrawal and π-donation introduced by Wiberg and Breneman in formamide.

Resonant inelastic X-ray scattering is a Raman spectroscopic technique that measures the energy and momentum transfer of a scattered X-ray photon to matter, providing detailed insight into the electronic structure of molecules and materials.^12^ RIXS exploits resonant enhancement of Raman scattering for X-ray photons and combines the element specificity of X-ray absorption with the ability to probe valence excited states over an exceptionally broad spectral range from the HOMO–LUMO gap to deep into the UV with contributions of a specific element.^13^

Fig. 1 shows the partial fluorescence yield (PFY) spectra of FA (blue), NMF (red) and DMF (green) which are essentially proportional to the X-ray absorption spectra of the nitrogen atoms. Bars indicate transitions calculated by time-dependent density functional theory in the restricted subspace approximation (RSA-TD-DFT, see ESI† for details).^14^ The lowest absorption line in this series is to the nitrogen 1s-to-LUMO transition with the LUMO being the π*-orbital of the N–C=O moiety. Notably, this line shifts to higher absorption energy with increasing methylation (grey area/line in Fig. 1) in the absence of C–N and C=O bond length changes (Table S1, ESI†). The Löwdin charges in Fig. 1 show a loss of negative charge at the nitrogen atom due charge withdrawal of the methyl groups (see charge analysis in ESI†). This valence charge reduction on the amide nitrogen reduces shielding of the N-1s core–hole upon X-ray absorption, yielding a higher energy of the nitrogen 1s-to-LUMO transition.

**Fig. 1:**
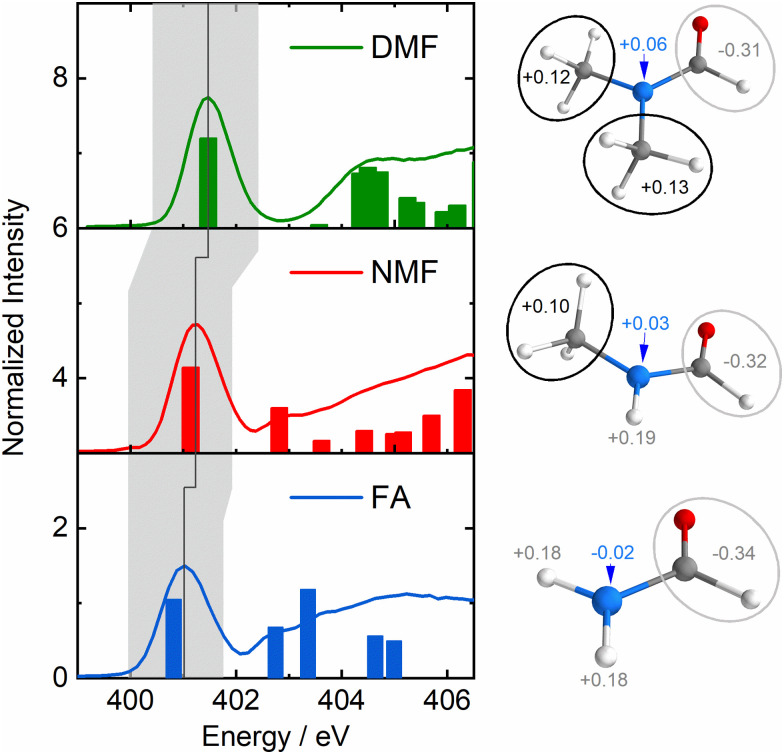
Left: Nitrogen-1s PFY spectra of formamide (FA, 1, blue), *N*-Methylformamide (NMF, 2 red) and *N*,*N*-dimethylformamide (DMF, 3, green) with calculated transitions as bars. The grey area highlights the nitrogen 1s-to-LUMO transition, the dark grey line indicates its shift to higher energy in the formamide series. Right: Löwdin charges of the nitrogen atom, the aldehyde group and the amide hydrogens/methyl groups.

The right side of Fig. 2 illustrates the RIXS experiment in an orbital picture. Red arrows indicate the absorption of an X-ray photon, promoting a nitrogen-1s electron to the π*-LUMO. The shift of the corresponding absorption line, *e.g.* nitrogen 1s-to-LUMO in Fig. 1, to higher energy signals an energy gap increase between two orbitals. The subsequent transition of an electron from the inner σ-orbital to the nitrogen-1s orbital that fills the core-hole (green arrows) effects the emission of a scattered X-ray photon and generates a final valence excited state of the molecules by having transferred the energy difference between incident and scattered X-ray photon – the energy transfer (dashed orange lines) – to the molecule.

**Fig. 2:**
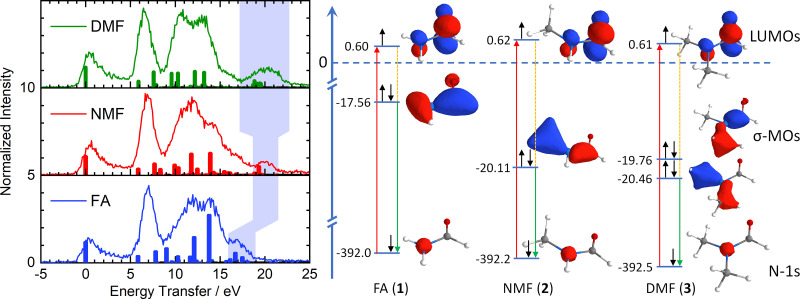
Left: Energy transfer spectra and calculated RIXS transitions (colour-coding of Fig. 1). The light blue area indicates excitations from the deep-lying σ framework, reflecting stabilization by methylation of the amide nitrogen. Right: Simplified orbital diagram with nitrogen 1s orbitals, π*-LUMOs and deep-lying σ-MOs and Kohn–Sham orbital energies. Red arrows: X-ray absorption. Green arrows: Resonant emission during inelastic scattering from deep-lying σ-MOs. Orange lines: Molecular excitations (shaded in light blue in left graph).

The left side of Fig. 2 shows the energy transfer spectra of FA, NMF and DMF for the lowest N-1s absorption resonance (Fig. 1) along with calculated Raman excitations for each molecule. The elastic scattering line coincides with 0 eV energy transfer. Vibrational progressions extend to nearly 5 eV and appear qualitatively very similar for all three formamides. These progressions are not included in our RSA-TD-DFT calculations^15^ since core-excited nuclear dynamics are not relevant to this study. Between 5 eV and 9 eV energy transfer, all three formamides have a pronounced feature. RSA-TD-DFT predicts this feature to stem from HOMO and HOMO−1 excitations in all three compounds. Theory underestimates the intensity of this peak strongly. This is likely due to the separation of π-states in the calculation. The accuracy of the modelled intensity for these final states depends on the choice of exchange correlation functional in the simulations (see ESI†) and may require a full vibrational treatment^16^ of the scattering process which is beyond the scope of this study.

All three formamides possess a broad feature between 9 and 16 eV which varies in shape and intensity. The RSA-TD-DFT simulations attribute this region to transitions from HOMO−2 to HOMO−5 orbitals in FA, to HOMO−8 in NMF and to HOMO−10 in DMF. More detailed information is difficult to extract in this region due to the high density of states. There is, however, a noteworthy insight: The HOMO−2 (FA, 1), HOMO−5 (NMF, 2) and HOMO−8 (DMF, 3) correspond to the respective primary π-orbitals. The calculated transitions from these π-orbitals shift to slightly higher energy in the methylation series due to hybridization with σ-orbitals of the methyl C–H bonds. We will return to the discussion of the π-orbitals later.

We now turn our discussion to the highest energy transfer region, marked by light blue shading in the experimental data of Fig. 2. Formamide features a set of low-intensity transitions in the form of a shoulder between 16 and 18 eV that shifts to higher energy transfer in NMF and becomes most pronounced in DMF. TD-DFT assigns this feature to inner valence excitations from the HOMO−6 in F, the HOMO−9 in NMF and a superposition of the HOMO−11 and HOMO−12 in DMF. Qualitatively, these orbitals are deep-lying σ-orbitals that comprise the X–N–Y moiety (X, Y = H, CH_3_) and define the primary bond strength of the central formamide framework. The shift of these σ-orbitals to higher binding energy signals stabilization of the entire σ-framework by 2.5 eV when a methyl group is introduced. Notably, this stabilization is already achieved by the substitution of one methyl group in NMF while an additional methyl group in DMF induces little further stabilization. A stabilization of lactams upon methylation of the amide nitrogen in the form of increasing basicity has been reported as early as 1960.^17^ Further clues towards a stabilizing effect in the formamide series were found in 1966 by UV spectroscopy and theoretical calculation.^11^

The simplified orbital diagram for the formamide series on the right side of Fig. 2 contains the nitrogen-1s orbitals, the π*-LUMOs and the inner σ-MOs along with the Kohn–Sham calculated energies. This diagram illustrates the stabilization of the deep-lying σ-framework as observed by nitrogen-1s RIXS: In formamide, the σ-orbital has an energy of −17.56 eV while it is stabilized by 2.55 eV in NMF (−20.11 eV). In DMF, two deep-lying σ-orbitals are found with −19.76 eV and −20.46 eV, amounting to a stabilization of 2.2 and 2.9 eV. On average, the stabilization of the σ-orbitals in DMF is identical to the one in NMF. The simulations reproduce the trend of the energetically highest experimental feature which broadens and strengthens without shifting energetically in DMF compared to NMF.

The shift of the highest energy transfer feature is direct experimental evidence that the deep-lying σ-framework is stabilized. The nature of this stabilization differs from hyperconjugation: The inner σ-orbital has no direct overlap with the π*-orbitals of the N–C=O moiety due to the planar configuration of the amides. Such an overlap would be required for hyperconjugation. Instead, there is significant net charge withdrawal from the nitrogen atom by the substituted methyl groups in NMF and DMF compared to the charge of the nitrogen atom in FA as partial charges in Fig. 1 indicate (see ESI† for details). This withdrawal occurs in part *via* the deepest σ-orbitals that are delocalized toward the methyl groups (Fig. 2), thereby stabilizing the deep-lying σ-framework of the amide moiety. The higher binding energy manifests in the observed strong shift of the highest energy transfer feature by several eV in NMF and DMF compared to FA.

Additionally, charge delocalization due to the methyl groups can be seen in the π-MOs in Fig. 3: The primary π-MO in FA (−11.68 eV) is fully delocalized over the N–C=O moiety with pure p-orbital character. In NMF, the lowest π-MO (−12.74 eV) is rehybridized and now involves the two appropriately aligned C–H σ-orbitals of the methyl group. In DMF, two C–H σ-orbitals per methyl group are hybridized with the N-2p_*z*_ orbital at −13.43 eV. Such hybridization is reminiscent of hyperconjugation but here, it involves C–H σ-orbitals and the N-2p_*z*_ orbital, all of which are bonding in character and fully occupied (with decreasing C=O π-character for increased methylation). The rehybridization of the π-MOs by methylation may also contribute to charge donation from the nitrogen atom to the methyl group(s). It leads to a stabilization of the deepest π-MO while destabilizing some of the π-MOs, manifesting in the changes of the RIXS signals in the energy transfer region at 10–15 eV.

**Fig. 3:**
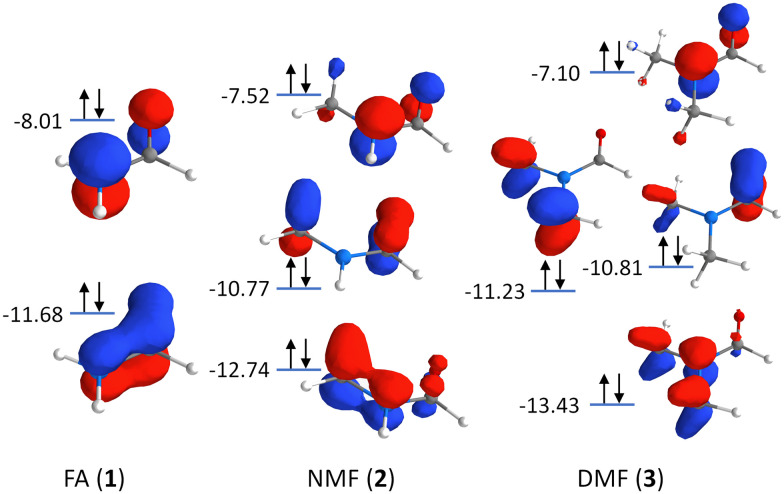
Orbital scheme depicting the π-MOs in the formamide series: Formamide (FA, 1), *N*-methylformamide (NMF, 2), and *N*,*N*-dimethylformamide (DMF, 3).

In conclusion, we have employed nitrogen-1s XAS & RIXS in combination with RSA-TD-DFT calculations to directly observe the valence electronic structure of the smallest formamides in solution. RIXS is uniquely positioned to detect shifts of specific charge density by small changes in the molecular composition such as the introduction of methyl groups, owing to the ability to measure deep-UV excitations in condensed matter. The stabilization of the σ-framework in the formamide series occurs upon introduction of a methyl group that is charge-withdrawing to the amide nitrogen. Introduction of a second methyl group in DMF does not lead to significant further stabilization. The evolution of transitions in the deep UV (>15 eV) upon methylation is direct experimental evidence of this stabilization by charge transfer from the amide nitrogen atom to the methyl groups. We conclude that this charge distribution mechanism is a major cause for the stability of secondary and tertiary amides upon introduction of an *R* group on the nitrogen atom.

We acknowledge funding by the ERC-ADG-2014 Advanced Investigator Grant no. 669531 EDAX *via* the Horizon 2020 EU Framework, Programme for Research and Innovation (A. F.), and the Deutsche Forschungsgemeinschaft (DFG, German Research Foundation) – SFB 925 – project 170620586 (M. O. and N. H.).

## Conflicts of interest

There are no conflicts to declare.

## Supplementary Material

CC-058-D2CC00053A-s001
