# Supplementary material for: R-Group stabilization in methylated formamides observed by resonant inelastic X-ray scattering
Source: Chem Commun (Camb). 2022 Jul 11;58(63):8834–7. doi: 10.1039/d2cc00053a (PMC9350990; doi:10.1039/d2cc00053a)
Supplement: CC-058-D2CC00053A-s001 [file CC-058-D2CC00053A-s001.pdf]

Electronic Supplementary Material (ESI) for Chemical Communications.
This journal is © The Royal Society of Chemistry 2022

R-Group Stabilization in Methylated Formamides observed by Resonant Inelastic X-ray Scattering

Miguel Ochmann[a], Vinícius Vaz da Cruz[b], Sebastian Eckert[b,*], Nils Huse[a,*] and Alexander Föhlisch[b,c]

SUPPORTING INFORMATION

Methods

Chemicals: Formamide (≥99,5 %, BioScience Grade), and N,N-Dimethylformamide (PEPTIPURE® ≥99,8 %) were purchased from Carl Roth Germany. N-Methylformamide (99%) was purchased from Sigma Aldrich Germany. All samples have been used without further purification. 500 mM sample solutions were prepared by diluting 9.9 mL formamide, 14.6 mL N-Methylformamide and 19.3 mL N,N-Dimethylformamide, respectively, to 500 mL with deionized water. The sample was flowed continuously at flow rates of ~0.45 mL/min, collected in a cold trap and discarded after single use.

Experimental Setup: Experiments were conducted at the U49-2_PGM-1 beamline of the storage ring facility BESSY II using the liquid flexRIXS experimental chamber.[1] Complementary measurements have been performed at the EDAX experiment at the UE49_SGM beamline. The sample was delivered by a cylindrical liquid jet with 20 µm diameter at $\sim 10^{-3}$ mbar pressure. Measurements were taken with horizontally polarized radiation with photon energies centred near the nitrogen K-edge (~400 eV) with a bandwidth of 250 meV. Resonant emission spectra were recorded in 90° scattering geometry using a modified Scienta XES 350 spectrometer.

Theoretical Calculations: The geometries of the three systems were optimized using the DFT module of the ORCA quantum chemistry package, version 4.20.[2] The PBE0 functional with the def2-TZVP(-f) basis set and the D3BJ correction were used. The RIJCOSX approximation was used with the def2/J auxiliary basis. Solvation in aqueous solution was treated implicitly using the conductor-like polarizable continuum model (CPCM) with the Gaussian charge scheme. Coordinates of all atoms and ground-state energies are given in Table S1. The RIXS simulations were performed using RSA-TD-DFT[3] and by considering transitions from all occupied valence orbitals as well as the nitrogen 1s orbital into the energetically lowest 20 unoccupied Kohn-Sham orbitals. The simulated transition energies for the X-ray absorption spectra were shifted by 11.125 eV to match the experimentally measured lowest nitrogen 1s-to-π* absorption lines in Fig. 1 of the main manuscript.

[a] *Department of Physics, University of Hamburg and Center for Free-Electron Laser Science, Luruper Chaussee 149, 22761 Hamburg, Germany. E-mail: nils.huse@uni-hamburg.de*
[b] *Institute for Methods and Instrumentation in Synchrotron Radiation Research G-ISRR, Helmholtz-Zentrum Berlin für Materialien und Energie, Albert-Einstein-Strasse 15, 12489 Berlin, Germany. E-mail: sebastian.eckert@helmholtz-berlin.de*
[c] *Institut für Physik and Astronomie, Universität Potsdam, Karl-Liebknecht-Strasse 24-25, 14476 Potsdam, Germany.*

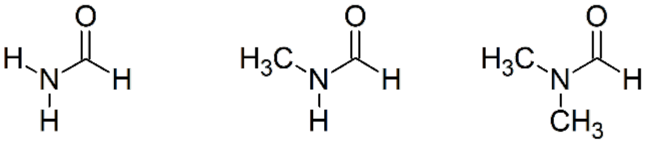

1 2 3

Scheme S1: Structural formulas of Formamide (1 = **FA**), N-Methylformamide (2 = **NMF**) and N,N-Dimethylformamide (3 = **DMF**).

The details of the RSA-TD-DFT modelling scheme are discussed by Vaz da Cruz et al.[3] Briefly, restriction of the donor and acceptor spaces in the TD-DFT simulations yields a set of orthogonal excited states, modelling excitations between the considered orbitals relevant for the considered RIXS processes. By generating the excited state pseudowavefunctions from the simulated TD-DFT amplitudes, the transition dipole matrix element between the states is calculated. These matrix elements are used in the Kramers-Heisenberg equation to simulate the spectral RIXS intensities. An orientational average of the RIXS intensities accounts for the isotropic distribution of molecular orientations in our solution experiment.

The comparison of simulated data from different exchange correlation functionals in Fig. S1 shows very similar RIXS spectra for each molecule, with the same spectral trends among the three investigated formamides for all investigated functionals. We have not simulated vibrational excitations which explains the absence of RIXS signals for 0 - 5 eV energy loss in the simulations. The main discrepancy between the measured and calculated RIXS spectra is found in the energy loss range of 5 - 9 eV. In particular, the strong experimental RIXS signal at 6 - 7 eV is too weak in the simulations. We suspect that this discrepancy is related to the disregard of vibrational motions during the RIXS process and the lack of explicit solvent modelling in our simulations. Emission lines at 9 - 16 eV energy loss have pi or non-bonding character and show the same spectral structure (with varying amplitude among the different functionals) that we find experimentally in this energy range. The double-peak structure in this energy loss range is, however, not as distinct as the simulations predict. Inclusion of vibrational progressions may account for the difference in this energy loss range. In summary, the spectral trends are consistent in the modelled spectra for all considered functionals, especially the transition trends related to the deep-lying sigma orbitals which are the focus of this study.

Table S1. Atomic coordinates of the optimized structures of the three investigated formamides.

**FA**	x / Å	y / Å	z / Å
C	-1.492616055	2.680886312	-0.226907554
O	-1.752033703	3.633916255	0.494053607
$H_{aldehyde}$	-0.409099751	1.909005268	-0.120091705
N	-0.409099751	1.909005268	-0.120091705
H_1	-0.261853588	1.142600655	-0.754778717
H_2	0.278330614	2.089385559	0.595052622

**NMF**	x / Å	y / Å	z / Å
C	-1.342224045	2.370107936	0.013356832
O	-1.437028601	3.484108497	0.516369091
$H_{aldehyde}$	-2.161287943	1.913764121	-0.565364612
N	-0.266023818	1.588750742	0.097440768
H_1	-0.299781773	0.687079867	-0.348770573
$C_{methyl2}$	0.928076094	1.981389823	0.810257742
$H_{methyl2a}$	1.679506592	1.204890215	0.684273046
$H_{methyl2b}$	0.724734931	2.109251411	1.875962979
$H_{methyl2c}$	1.319478563	2.922387387	0.418784726

**DMF**	x / Å	y / Å	z / Å
C	-1.211461355	2.428266458	-0.205106303
O	-1.138150348	3.649268074	-0.111256596
$H_{aldehyde}$	-2.064109102	1.933620417	-0.697699479
N	-0.311573354	1.552139632	0.253534972
$C_{methyl1}$	-0.498213452	0.130171448	0.091310811
$H_{methyl1a}$	1.770670416	1.63661476	0.394312078
$H_{methyl1b}$	0.912036976	1.583684634	1.946930407
$H_{methyl1c}$	0.893016476	3.074633445	0.97246584
$C_{methyl2}$	0.885687851	1.988005099	0.931482070
$H_{methyl2a}$	0.325846595	-0.298574897	-0.485263320
$H_{methyl2b}$	-1.433618123	-0.058508137	-0.434929436
$H_{methyl2c}$	-0.53405258	-0.362980933	1.066428955

	**FA**	**NMF**	**DMF**
C=O / Å	1.22284446	1.22597165	1.226795508
C–N / Å	1.33462263	1.33259000	1.3370671

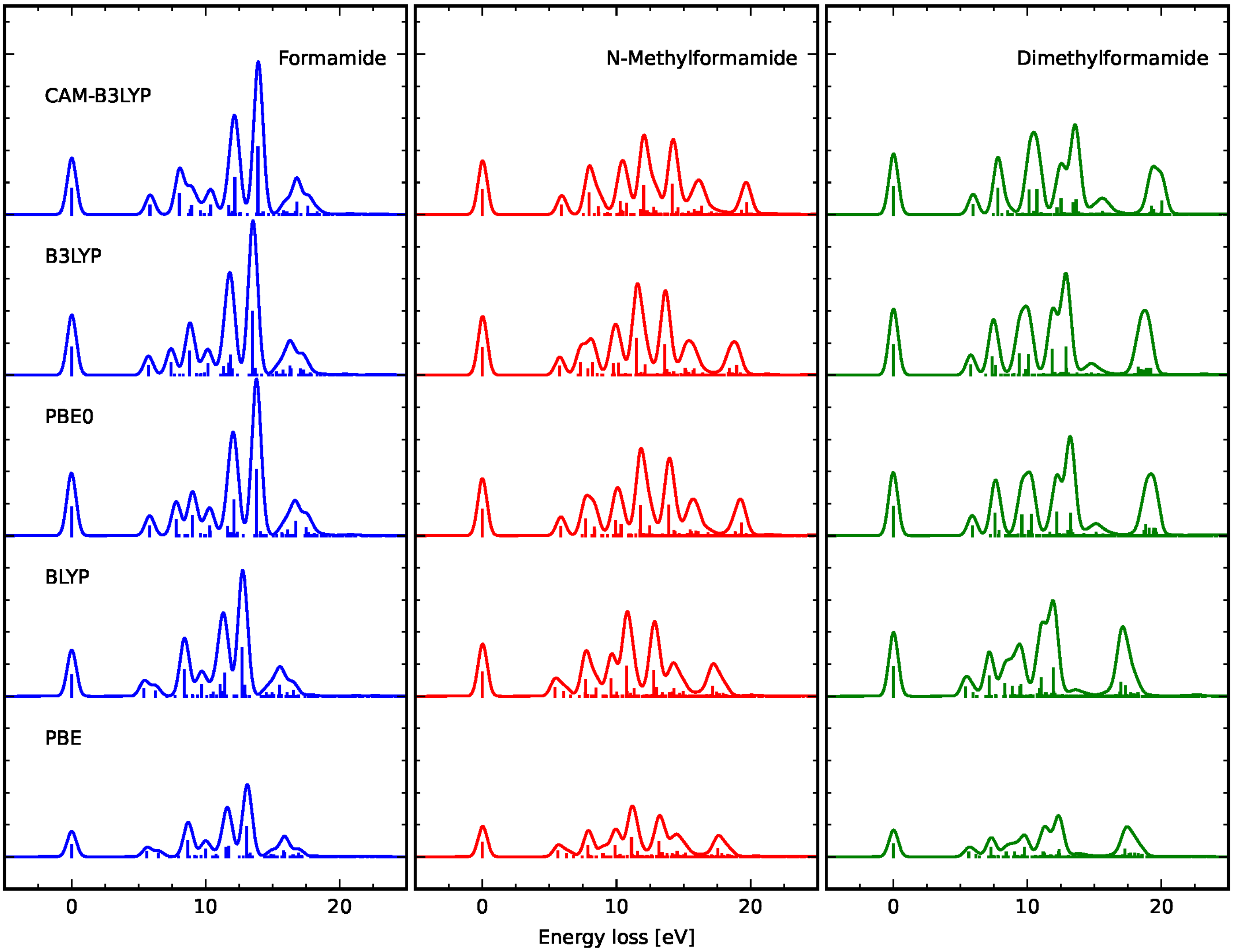

Figure S1. Comparison of RIXS spectra for formamide (blue), N-methylformamide (red) and dimethylformamide (green) generated by RSA-TD-DFT with the indicated functionals.

Charge Analysis: We have also analysed the partial charges in our modelling approach by calculation of four different charge measures according to Löwdin, Mulliken, Hirshfeld and natural population analysis (NPA). The result of this analysis is displayed in Fig. S2. We would like to point out that the y-axis runs from higher at the bottom to lower values at the top such that a loss of electronic charge density (and therefore a mathematically higher charge value) corresponds to a decreasing graph with increased methylation.

While the sum of partial charges of the aldehyde group appears to change very little, all charge measures show the same trend of decreasing electronic charge at the nitrogen atom and of increasing electronic charge for the sum of partial charges on the amine hydrogen atom(s) and methyl group(s). We have summed the partial charges for the amine hydrogen atom(s) and methyl group(s) to consistently quantify the charge on these substituents. Most notably, the methyl groups effectively gain charge while the partial charge on the amine hydrogen atom does not change appreciably when the first methyl group is introduced as detailed in Table S1.

The methyl group experiences a net increase in charge as electronic charge density is delocalized away from the nitrogen atom onto methyl group(s) by means of rehybridization of the deepest σ-orbitals and the deepest π-orbitals (Fig. 2 and Fig. 3 in the main manuscript, respectively). Interestingly, the deepest π-orbitals of NMF and DMF in Fig. 3 are clearly hybridized with the σ-orbitals of two appropriately aligned C-H bonds in each of the methyl groups, reminiscent of orbital hybridization in hyper-conjugation, yet involving fully occupied valence orbitals.

Our charge analysis is fully consistent with the experimentally observed shift of the lowest nitrogen-1s excitation to higher energy in Fig. 1 of the main manuscript. The chemical shift is due to a decrease of electronic valence charge density on the nitrogen atom. It is commonly referred to as core-level shift in the literature and has often been used in assessing systematic changes of charge delocalization (e.g. by ligand binding) or charge transfer (e.g. an oxidative shift).[4,5]

The transfer of charge toward the methyl groups may appear surprising as methyl groups are usually conceived as electron donors when attached to another carbon atom. However, such a generalization does not hold for atoms of other elements.[6,7]

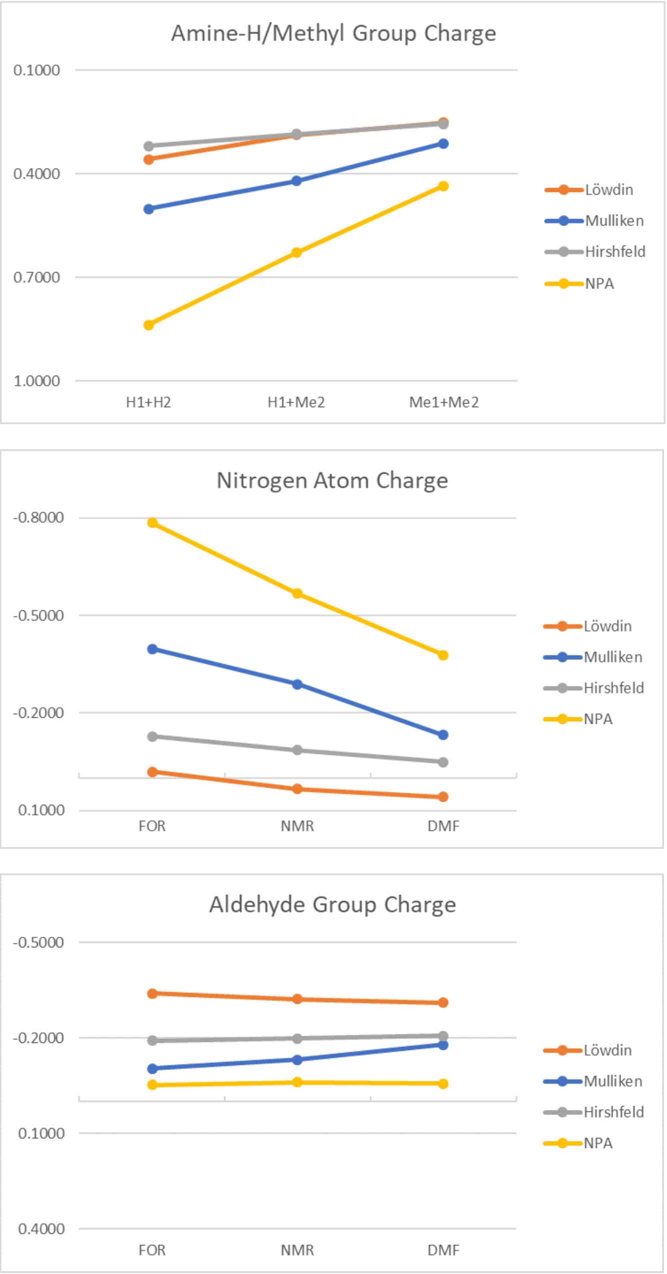

Figure S2: Trends in partial charges of the amine hydrogen atoms/methyl groups (top), the nitrogen atom (middle) and the aldehyde group (bottom) upon single and double methylation of formamide according to the four indicated methods. The charge range is set to 0.9e, and the y-axis runs from higher values (bottom) to lower ones (top).

Table S1: Partial charges of atoms and chemical groups as calculated by to the four indicated wavefunction-based methods. The amine hydrogen atoms in **FOR** and **NMF** are termed H_1 and H_2, the methyl groups are termed Me_1 and Me_2.

		**Löwdin**	**Mulliken**	**Hirshfeld**	**NPA**
**FA**	C	-0.3078	0.4036	0.1497	0.4932
	O	-0.1468	-0.6047	-0.3973	-0.6743
	$H_{aldehyde}$	0.1152	0.0965	0.0559	0.1282
	N	-0.0191	-0.3970	-0.1275	-0.7848
	H_1	0.1764	0.2426	0.1564	0.4162
	H_2	0.1821	0.2590	0.1628	0.4216
	Sum	0.0000	0.0000	0.0000	0.0000
	Aldehyde	-0.3393	-0.1046	-0.1917	-0.0530
	$H_1 + H_2$	0.3584	0.5016	0.3192	0.8378
**NMR**	C	-0.2936	0.3831	0.1422	0.4913
	O	-0.1469	-0.6202	-0.3949	-0.6849
	$H_{aldehyde}$	0.1186	0.1047	0.0537	0.1328
	N	0.0341	-0.2887	-0.0862	-0.5677
	H_1	0.1946	0.2456	0.1605	0.4248
	$C_{methyl2}$	-0.2606	-0.2803	-0.0135	-0.4433
	$H_{methyl2a}$	0.1223	0.1610	0.0565	0.2256
	$H_{methyl2b}$	0.1155	0.1477	0.0409	0.2096
	$H_{methyl2c}$	0.1160	0.1471	0.0407	0.2118
	Sum	0.0000	0.0000	0.0000	0.0000
	Aldehyde	-0.3220	-0.1324	-0.1990	-0.0608
	$H_1 + Me_2$	0.2879	0.4211	0.2851	0.6285
**DMF**	C	-0.2802	0.3516	0.1376	0.4998
	O	-0.1519	-0.6274	-0.3950	-0.6872
	$H_{aldehyde}$	0.1218	0.0970	0.0503	0.1311
	N	0.0584	-0.1328	-0.0488	-0.3785
	$C_{methyl1}$	-0.2351	-0.3102	-0.0157	-0.4411
	$H_{methyl1a}$	0.1178	0.1492	0.0467	0.2084
	$H_{methyl1b}$	0.1242	0.1536	0.0545	0.2240
	$H_{methyl1c}$	0.1178	0.1496	0.0467	0.2084
	$C_{methyl2}$	-0.2340	-0.2870	-0.0092	-0.4225
	$H_{methyl2a}$	0.1197	0.1510	0.0469	0.2116
	$H_{methyl2b}$	0.1197	0.1513	0.0469	0.2115
	$H_{methyl2c}$	0.1220	0.1542	0.0391	0.2347
	Sum	0.0000	0.0000	0.0000	0.0000
	Aldehyde	-0.3104	-0.1788	-0.2071	-0.0563
	$Me_1 + Me_2$	0.2520	0.3116	0.2559	0.4349

References

1 K. Kunnus, I. Rajkovic, S. Schreck, W. Quevedo, S. Eckert, M. Beye, E. Suljoti, C. Weniger, C. Kalus, S. Grübel, M. Scholz, D. Nordlund, W. Zhang, R. W. Hartsock, K. J. Gaffney, W. F. Schlotter, J. J. Turner, B. Kennedy, F. Hennies, S. Techert, P. Wernet and A. Föhlisch, *Rev. Sci. Instrum.*, 2012, **83**, 123109.

2 F. Neese, *WIREs Comput. Mol. Sci.*, 2012, **2**, 73–78.

3 V. Vaz da Cruz, S. Eckert and A. Föhlisch, *Phys. Chem. Chem. Phys.*, 2021, **23**, 1835–1848.

4 D. Nolting, E. F. Aziz, N. Ottosson, M. Faubel, I. V. Hertel and B. Winter, *J. Am. Chem. Soc.*, 2007, **129**, 14068–14073.

5 J. M. García-Lastra, P. L. Cook, F. J. Himpsel and A. Rubio, *J. Chem. Phys.*, 2010, **133**, 151103.

6 F. Teixidor, G. Barberà, A. Vaca, R. Kivekäs, R. Sillanpää, J. Oliva and C. Viñas, *J. Am. Chem. Soc.*, 2005, **127**, 10158–10159.

7 Q. Li, N. Wang and Z. Yu, *J. Mol. Struct. THEOCHEM*, 2008, **862**, 74–79.
